# Laboratory diagnosis for Covid-19: A mini-review

**DOI:** 10.1590/0037-8682-0451-2020

**Published:** 2020-08-26

**Authors:** Juliana Lemos Dal Pizzol, Vanusa Pousada da Hora, Ana Júlia Reis, Júlia Vianna, Ivy Ramis, Andrea von Groll, Pedro Almeida da Silva

**Affiliations:** 1 Universidade Federal do Rio Grande, Núcleo de Pesquisa em Microbiologia Médica, Programa de Pós-Graduação em Ciências da Saúde, Rio Grande, RS, Brasil.

**Keywords:** COVID-19, Diagnostic, Serology, Molecular, Biomarkers

## Abstract

Coronavirus disease (COVID-19) is a pandemic caused by a new coronavirus, called SARS-CoV-2. This disease was first identified in December 2019 and rapidly developed into a challenge to the public health systems around the world. In the absence of a vaccine and specific therapies, disease control and promotion of patient health are strongly dependent on a rapid and accurate diagnosis. This review describes the main laboratory approaches to making a diagnosis of COVID-19 and identifying those previously infected with SARS-CoV-2.

## INTRODUCTION

Rapid and accurate diagnosis of Coronavirus disease (COVID-19) is essential for pandemic control as well as for establishing an adequate therapeutic strategy to reduce morbidity and mortality. For both epidemiological and clinical purposes, several methodological approaches have been developed. In this article, we will cover the main laboratory methods and protocols that have been used for the control and management of COVID-19.

## USING LABORATORY DIAGNOSIS TO ENHANCE THE CONTROL OF COVID-19

Reliable laboratory diagnosis represents one of the main tools for the promotion, prevention, and control of infectious diseases^1^. The diagnostic methods for COVID-19 fall under two main categories: immunological and molecular. Immunological tests can be serological tests that mainly detect antibodies in blood or viral antigens in respiratory secretions, and both can be performed with point-of-care platforms. Regarding molecular tests, they are based on the detection of SARS-CoV-2 RNA mainly in nasopharyngeal samples, which in most cases require adequate laboratory infrastructure. In addition to the cited tests, other laboratory parameters have been used as an aid in the clinical monitoring of patients with COVID-19^2^ ^-^ ^4^. 

## SEROLOGICAL TESTS

Serological tests are especially important for the diagnosis of patients with mild to moderate disease, in the absence of molecular diagnostics^5^. These tests can have several benefits, such as estimating the transmissibility and lethality rates, assessing individual and community immunity, and valuing the need and effectiveness of nonpharmaceutical interventions (e.g., social isolation). Furthermore, the plasma of convalescents with high levels of antibody production could be used as a therapeutic support^6^. Several serological tests based on enzyme-linked immunosorbent assay (ELISA), and lateral flow immunochromatography (LFI) devices have been developed by different companies worldwide. IgM and IgG antibodies detected on ELISA have more than 95% specificity in the diagnosis of COVID-19 (18). High titers of IgG antibodies detected by ELISA demonstrate a positive correlation with neutralizing antibodies^7^.

Given their point-of-care characteristics, LFI platforms have been widely used. In general, this method detects IgM and IgG antibodies in approximately 20 minutes, individually or simultaneously. Antibodies to glycoprotein S (spike) are analyzed from blood samples obtained by finger puncture without the need for sophisticated equipment or specialized professionals^8^. However, these tests are purely qualitative and can only indicate the presence or absence of SARS-CoV-2 antibodies^5^. Despite its potential value as a tool for pandemic control, the validation of LFI tests remains challenging^9^. The ability to assess their accuracy (sensitivity and specificity) as well as their ability to monitor immunity over time remains insufficient^10^. 

Another matter of concern is inappropriate interpretation of the result, such as a false understanding that a positive result indicates immunity against the SARS-CoV-2, whereas a positive result on the serological test indicates that the person has come into contact with the virus and developed antibodies, but it is not clear whether these antibodies will provide protection against a reinfection^11^.

Currently, antibody responses against SARS-CoV-2 remain poorly understood, and the clinical usefulness of the serological test is still unclear^12^. Although the detection of IgM and IgG by ELISA is positive even on the fourth day after the onset of symptoms, high levels of these antibodies are produced in the second and third weeks of the disease^5^. From the time of onset, the IgM antibody titer increases; 2 weeks after the onset of symptoms, both IgG and IgM are present and their levels start to decrease after the fourth week. IgM is notoriously nonspecific, and because it takes weeks to develop specific IgG responses, serological detection is unlikely to play an active role in case management, with diagnosis/confirmation of late cases of COVID-19 or determining the immunity of health professionals being the exceptions^12^. The acute antibody response to SARS-CoV-2 in 285 patients in China’s Hubei province was detected using a chemiluminescence immunoenzymatic test (CLIA). The result showed that the proportion of patients positive for specific IgG reached 100% approximately 17 to 19 days after the onset of symptoms. Meanwhile, the proportion of patients with specific IgM reached 94.1% at 20 to 22 days after the onset of symptoms. Seroconversion to IgG and IgM occurred simultaneously or sequentially, giving an average seroconversion time of 13 days after the onset of symptoms. The study data indicate that serological tests can be complementary, especially in the diagnosis of suspected patients with negative molecular results and also in the search for asymptomatic infections among close contacts^13^.

An interesting aspect is that the most severe cases have higher levels of IgM and IgG in than the mild cases^14^ ^,^ ^15^. In this context, the quantitative detection of antibodies can be an important aid in clinical practice^16^.

It is worth mentioning that the majority of immunoassays for SARS-CoV-2 have immunogenic proteins as their main target: 1) protein S (spike), which is the most highly exposed viral protein, and 2) nucleocapsid protein (N), which is abundantly expressed during infection^17^. Normally, most antibodies are produced against the most abundant protein present in the virus (N). Therefore, tests that detect antibodies to N would be the most sensitive. However, the protein S receptor binding domain (RBD-S) is the host's attachment protein, and antibodies to RBD-S would be very specific and are expected to be neutralizing. In this case, the use of one or both antigens to detect IgG and IgM would result in high sensitivity^5^ ^,^ ^7^. 

## MOLECULAR TESTS

Most molecular tests, unlike serological tests, are performed in a specialized laboratory using cutting-edge equipment and highly qualified staff, so their use is limited. Nasopharyngeal (NP) swabs are considered the standard samples for the detection of SARS-CoV-2. In addition to the NP swabs, the use of samples from the lower respiratory tract (sputum or bronchial lavage) and oropharyngeal (OP) swabs are used as alternatives to improve the biosafety of health care workers^18^. A robust study on the detection of SARS-CoV-2 in different clinical samples involved 205 hospitalized patients from three Chinese hospitals, with a total of 1,070 samples collected. The NP swabs had the highest viral load, although at the time of collection, the stage of disease or the associated clinical history was not available for many of these patients^2^ ^,^ ^19^. For the detection of SARS-CoV-2 by this technique, samples must be collected when the patient is in the acute phase of infection, preferably up to 5 days after the onset of symptoms^20^. This method has the advantage of being both quantitative and highly specific^18^. 

Reverse transcription followed by real-time reverse transcription polymerase chain reaction (RT-PCR) is considered the gold standard for the diagnosis of COVID-19. The first protocol recommended by the WHO was published by Charité Institute, Berlin University, Germany^21^. It is based on TaqMan technology, with indicated primers and probes to detect the RNA-dependent RNA polymerase (RdRp), envelope protein (E), and nucleocapsid protein (N) genes. Subsequently, several in-house methods have been reported by the WHO, and they are being validated in WHO partner laboratories (Table 1).

**TABLE 1:** List of RT-PCR protocols indicated by the WHO.

Institute	Gene targets
China CDC, China	ORF1ab and N
Institute Pasteur, France	Two targets in RdRP
US CDC, USA	Three targets in N gene
National Institute of Infectious Diseases, Japan	Pancorona and multiple targets, Spike protein
Charité, Germany	RdRP, E, N
HKU, Hong Kong SAR	ORF1b-nsp14, N
National Institute of Health, Thailand	N

(Source: https://www.who.int/who-documents-detail/molecular-assays-to-diagnose-COVID-19-summary-table-of-available-protocols)

False-negative results can occur mainly because of inadequate extraction of nucleic acid; poor sample quality; low viral load; sample collection time; incorrect sample storage, transportation, and handling; and PCR inhibition^22^ ^-^ ^24^. 

Results of different RT-PCRs protocols have shown variation in their performance depending on the primers and probes. One comparative study of the sensitivity of SARS-CoV-2 RT-PCR tests developed by Charité (Germany), HKU (Hong-Kong), China CDC (China), US CDC (United-States), and Institut Pasteur, Paris (France) showed that all RT-PCR assays performed well for SARS-CoV-2 detection, but the authors pointed out that the assays of RdRp Institut Pasteur (IP2, IP4), N China CDC, and N1 US CDC were the most sensitive^25^. 

The use of specific primers determines the high specificity of RT-PCR, and the possibility of false-positives cannot be excluded. In this sense, a negative template control should be introduced in every RT-PCR^26^. A chest computerized tomography (CT) scan can be used as a complementary diagnostic tool that enables physicians to effectively detect COVID-19 infection in several RT-PCR false-negative cases. Repeat tests can be particularly important if the patient has a clinical picture of viral pneumonia, history of exposure, and/or radiographic findings (CT or magnetic resonance imaging) compatible with COVID-19 pneumonia^12^.

The development of new diagnostic platforms can improve molecular methods in terms of speed, sensitivity, and accessibility in the diagnosis of COVID-19. Currently, approximately 11 molecular devices have received urgent approval from the National Administration of Medical Products in China^18^. In addition, automated RT-PCR systems have been developed, such as the Xpress SARS CoV-2 Test (Cepheid, USA). Using the GeneXpert platform, SARS-CoV-2 E and N2 genes can be detected in approximately 45 minutes. Another innovative alternative is the Abbott ID Now COVID-19 handheld instrument, which detects the SARS-CoV-2 RdRp and N genes. Both Xpress SARS-CoV-2 and Abbott ID Now COVID-19 were authorized for emergency use by the Food and Drug Administration (FDA), USA^27^ ^,^ ^28^.

However, with the global shortage of kits, many countries have begun to carry out in-house RT-PCR to overcome this shortage. The most frequently used in-house protocols are as follows: 1) Hospital Charité - University of Berlin that targets the genes E, N and RdRp^21^, endorsed by the WHO, 2) CDC-China, which targets the ORF1ab and N genes (CDC-CHINA 2020), and 3) CDC-USA, which uses three targets within the N gene^29^.

Another molecular approach that may be useful, especially in places where there is no need for expensive thermocycling equipment is reverse transcription loop-mediated isothermal amplification (RT-LAMP) using the SARS-CoV-2 spike ORF1ab and S genes^30^ ^,^ ^31^. In addition, the use of biosensors to detect SARS-CoV-2 viral RNA has also been tested. The newly developed biosensor integrates the plasmatic photothermal effect and plasmon resonance detection transduction. Validity and selectivity were determined by using the SARS-CoV-2 RdRp and ORF1ab sequences as targets^32^.

BIOSAFETY

Laboratory testing is a process divided into three main phases: preanalytical, analytical, and postanalytical. In the preanalytical stage, individual protection measures in relation to sample collection are fundamental to good biosafety practices. Based on the WHO guidelines, all clinical samples should be considered potentially infectious, and health professionals must use suitable and a complete set of personal protective equipment (PPE) when obtaining/handling patient samples. Normally, the use of a disposable apron, gloves, bonnet, foot protection, protective goggles, and an N95 breathing mask is expected.

Clinical samples must be transported to the laboratory in packaging appropriate for level-2 biological risk inside a leak-proof cryogenic box and with a clearly visible biohazard label^33^. The samples can be stored at 2-8°C for up to 72 hours after collection. If the sample needs to be stored for long, it must be done at a temperature of at least -70°C. The extracted nucleic acid must also be stored at the same minimum temperature of -70°C^29^.

Sample processing must be done inside a class-2 biological cabin using suitable clothing and a complete set of PPE. All surfaces, cryo-boxes, and equipment must be cleaned using 0.1% sodium hypochlorite, 62-71% ethanol, 0.5% hydrogen peroxide, and quaternary ammonium or phenolic compounds^34^.

## NON-SPECIFIC LABORATORY TESTS

Results of laboratory tests can increase the support for the diagnosis, prognosis, and monitoring of patients through the detection and measurement of different biomarkers. Although nonspecific, some biomarkers have been reported to be associated with the infectious process of SARS-CoV-2. Therefore, low lymphocyte and platelet counts; low serum albumin levels; and increased serum levels of C-reactive protein, D-dimer, ferritin, lactate dehydrogenase, transaminases, and interleukin-6 can be used in risk stratification to predict the severity of COVID-19^35^ ^-^ ^39^. In addition, cytokine storms with high levels of IL-2R, IL-6, IL-10, and TNF-α and a reduction in the absolute numbers of CD4 + and CD8 + T lymphocytes have been related to severe cases of COVID-19^40^, with progression to cardiovascular collapse, multiple organ failure, and rapid deaths^41^.

## DISCUSSION

This review provides an overview of the diagnostic methods used for COVID-19. New studies on this topic are rapidly becoming available in the literature, but there are crucial gaps that prevent an effective response in this pandemic. Although RT-PCR is the most widely used option for diagnosis, it can provide false-negative results, and its use is limited by the requirement of laboratory infrastructure and high costs^22^ ^,^ ^42^ ^,^ ^43^. Molecular point-of-care tests, with high precision, that present fast results, low cost, and easy execution are urgently needed to expand the diagnostic capacity of health systems, especially in low-income settings.

Serological tests can function as complementary tools for the diagnosis of COVID-19. Despite the fact that positive serological results can be obtained around 4-7 days after the onset of the symptoms, their usefulness for patients with viral loads below the detection limit of real-time RT-PCR assays is questionable^19^. These tests are also important to understand the epidemiology of SARS-CoV-2, including the role of asymptomatic infection and current or past infection^44^.

Although diagnosis by real-time RT-PCR is still essential to identify acute SARS-CoV-2 infection, serological tests and epidemiological data are necessary to understand past pandemics and predict their future^9^ ^,^ ^10^. The Global Health Security (GHS) index shows that only 19% of the countries have the capacity to detect and report epidemics of potential international concern, with fewer than 5% of the countries having the ability to respond quickly and mitigate the spread of an epidemic. In this sense, it is clear that no country has shown total preparedness for epidemics or pandemics^45^.

The global spread of COVID-19 should help nations establish new and fundamental priorities in the field of research and public health policies. The costs of lives lost and economic crises should serve as stimuli, especially for emerging economies, so that industrial-scale production in the health sector is accelerated and able to meet national requirements. 
